# The associations of muscle mass with glucose and lipid metabolism are influenced by body fat accumulation in children and adolescents

**DOI:** 10.3389/fendo.2022.976998

**Published:** 2022-09-15

**Authors:** Liwang Gao, Hong Cheng, Yinkun Yan, Junting Liu, Xinying Shan, Xi Wang, Jie Mi

**Affiliations:** ^1^ Center for Non-communicable Disease Management, Beijing Children’s Hospital, Capital Medical University, National Center for Children’s Health, Beijing, China; ^2^ Department of Epidemiology, Capital Institute of Pediatrics, Beijing, China

**Keywords:** obesity, muscle mass and fat mass, glucose, lipids, children

## Abstract

**Objective:**

To evaluate the prevalence of hyperglycemia and dyslipidemia among different body composition and investigate the associations of body composition indicators, especially the muscle mass, with glucose and lipids metabolism in children and adolescents.

**Methods:**

This nationwide cross-sectional study included 8,905 children and adolescents aged 6 to 18 years. All participants underwent dual-energy x-ray absorptiometry and their blood-concentrated glucose and lipids (including TC, TG, LDL-C and HDL-c) were measured. Mixed model, hierarchical analysis, and piecewise regression were used to study the effect of body composition indicators, especially the muscle mass, on glucose and lipids metabolism.

**Results:**

The greatest prevalence of high total cholesterol (TC, 6.9% and 6.9%) and high triglyceride (22.3% and 6.6%) was found in both boys and girls with high muscle mass and high fat mass, and girls with high muscle mass and high fat mass also had the highest prevalence of hyperglycemia (7.1%). After fat stratification, higher muscle mass was associated with lower odds of hyperglycemia (OR = 0.62; 95%CI: 0.46,0.84; *P =* 0.002) and muscle mass was inversely associated with TC (β = −0.07; 95%CI: -0.12,-0.03; *P <* 0.001) in boys with normal fat mass, but high muscle mass was not significantly associated with hyperglycemia and TC in high-fat-mass group (*P* = 0.368 and 0.372).

**Conclusions:**

The body composition phenotype of high muscle and high fat mass have the highest prevalence of dysglycemia and dyslipidemia. Higher muscle mass was associated with a lower risk of hyperglycemia and TC levels in individuals only with normal fat mass.

## Introduction

With the dramatic increase in childhood obesity globally and in China, the risk of abnormal glucose and lipid metabolism in the early stages of the cardiovascular disease chain is expected to accelerate (1–3). Despite the strong association between higher body mass index (BMI) and abnormal glucose and lipid metabolism (4, 5), obesity defined by BMI is known as a heterogeneous condition with different cardiometabolic risk among same-weight status (6).

BMI is limited by not discriminating body composition, and individuals with the same BMI status have broad variations in body composition (7, 8). This is important because fat and muscle confers different cardiovascular risk (9). Determining the effect of body composition on the development of abnormal glucose and lipid metabolism can help to understand the mechanism of obesity-induced cardiometabolic abnormalities and prevent cardiovascular disease (10). Previous studies have reported the impact of accumulation of fat in relation to glucose and lipids metabolism (11, 12), and review articles have investigated the association of muscle mass with metabolic abnormalities (13). However, the above articles separately studied the effects of fat or muscle on glucose and lipid metabolism, and whether body composition would affect glycolipid metabolism in children and adolescents remains unclear. In addition, the association of muscle mass with metabolic abnormalities is inconsistent, which may be related to inconsistent body composition measurement techniques and failure to control for potential biological and lifestyle confounders (13).

Furthermore, it has been found that fat has a negative effect on muscle in children (14, 15). The increase in body fat mass, especially the increase of fatty acids and inflammatory molecules in visceral adipose tissue, can induce muscle inflammation and negatively regulate muscle cell metabolism (16). But it is not known whether the association of muscle mass with glucose and lipids metabolism is consistent in normal-fat mass and high-fat mass. Based on that, we hypothesized that different body composition has different risk of abnormal glucose and lipid metabolism, and the protective effect of muscle on glucose and lipid metabolism is affected by body fat mass accumulation.

Therefore, we evaluated the prevalence of hyperglycemia and dyslipidemia in different body compositions and examined associations of body composition [assessed by whole-body dual-energy X-ray absorptiometry (DXA)] with glucose and lipids metabolism in large-scale cross-sectional data of children and adolescents aged 6-18 years, especially focusing on the effect of muscle mass.

## Methods

### Study participants

The present study used data from the China Child and Adolescent Cardiovascular Health (CCACH) study, a nationally cross-sectional survey conducted between January 1, 2013, and December 31, 2015. The CCACH study, which included four cities in the northern region (Beijing, Changchun, Jinan, and Yinchuan) and three cities in the southern region (Shanghai, Chongqing, and Chengdu) of China, was designed to examine the cardiovascular health of school-age children and adolescents in China; the details of the study methods have been published elsewhere (17, 18). To assess the relationship between body composition and glucose and lipids metabolism, the data from 11,169 participants who underwent DXA were examined. Among them, those with missing data for glucose and lipids (n = 2,264) were excluded from the analyses. Ultimately, 8,905 participants were included in the analyses.

This study protocol was approved by the ethics committee of the Capital Institute of Pediatrics (approval number: 2012062), and written informed consent was obtained from all participants and/or their parents.

### Whole-body DXA scan

Body composition was measured using whole-body DXA (Hologic Explorer, Hologic Inc., Bedford, MA, USA) by a trained technician according to a standardized protocol. Before the DXA scan, the phantom was verified every other day according to the manufacturer’s guidelines, and participants were required to wear light clothing and remove all metal jewelry, plastic, and rubber materials. Fat mass index (FMI) was calculated as whole-fat mass (kg) divided by the square of height (m^2^), and muscle mass index (MMI) was calculated as whole lean mass (kg) excluding bone mineral content (kg) divided by height squared (m^2^).

### Anthropometric measurements

Wall-mounted stadiometers and Beam scales were used to measure standing height and weight, respectively. Participants were barefoot during height measurement, and they were asked to remove any heavy clothing during weight measurement. Waist measurements are available elsewhere (19).

### Biochemical measurements

After an overnight fast (at least 10 hours), venous blood samples were collected by direct venipuncture into ethylene diamine tetraacetic acid (EDTA) anticoagulant tube, after which they were centrifuged for 10 minutes at 1509.3 × g at 4°C. The plasma samples from each study site were transported by air in the form of dry ice preservation to the central clinical laboratory of the Capital Institute of Pediatrics, where the specimens were stored at -80°C until the laboratory tests. Fasting plasma glucose (FPG) (enzyme hexokinase method) and lipids (enzymatic methods or direct method) were measured using an autoanalyzer (Hitachi 7080, Tokyo, Japan). Fasting insulin was determined by RIA (Cisbio Bioassays), which had no cross-reactivity with proinsulin. All the intra- and inter-assay coefficients of variation were <5% and <10%, respectively.

### Definitions

The sex- and age-specific BMI (weight in kilograms divided by height in meters squared) cutoff values recommended by the International Obesity Task Force were used to define weight status (normal weight, overweight, obesity) (20). Hyperglycemia was defined as FPG ≥ 6.1 mmol/L or taking antihyperglycemic medications (21). Insulin resistance was defined following the World Health Organization as values in the highest quartile of homeostasis model assessment of insulin resistance (HOMA-IR) (21), which was calculated with the following formula: fasting insulin (mU/L) × FPG (mmol/L)/22.5. Dyslipidemia, including high TC, high TG, high low-density lipoprotein cholesterol (LDL-C), low high-density lipoprotein cholesterol (HDL-C), and high non-HDL-C, was defined by the sex and age-specific lipoprotein cutoffs based on Chinese children and adolescents (22), or taking lipid-lowering medications.

### Covariates

For statistical analyses, the following data were further collected: resident region, family income, puberty development (menarche/spermarche), behaviors and habits in the past six months (physical activity, sedentary activity, drinking alcohol, and smoking status). A standard self-administered questionnaire was used to collect the above information. Subjects mainly answered questions by themselves or were assisted by their parents/guardians when necessary. The sedentary activity was defined as the total time spent watching TV and playing computer games < 120 min/d. Physical activity was defined as moderate or vigorous physical activity ≥ 60 min/d. Alcohol drinking was defined as consumption of alcohol ≥18 g during the past month. Smoking was defined as having smoked or attempting to smoke in the past month.

### Statistical analyses

Continuous variables were expressed as mean ± standard deviation (SD) or medians (IQRs) according to their distribution type, and categorical variables were displayed as the frequency with percentage. TG was log-transformed before analysis because of the non-normal distribution. ANOVA, Kruskal-Wallis test, and χ2 tests were conducted to compare the general characteristics among the body composition. FMI and MMI were standardized to a mean of 0 and an SD of 1 specific for sex and age before analysis.

A surface plot fitted by the Gaussian method was used to illustrate the distribution of glucose and lipids on body composition. A generalized additive model (GAM) with quadratically penalized spline terms was applied to check the non-linear relations between muscle mass and glucose and lipids in children and adolescents with different fat masses. Multivariable-adjusted linear regression coefficients (βs) and odds ratios (ORs) and their 95%CIs for glucose and lipids per 1-SD increase in muscle mass were estimated by hierarchical analysis and piecewise regression to assess the associations between muscle mass and glucose and lipids in individuals with different fat mass, adjusting for family income, puberty development, physical activity, sedentary activity, drinking alcohol and smoking status.

Furthermore, to test the association between body composition and glucose and lipids, we divided participants into 4 phenotype: Normal muscle-normal fat mass: MMI z-score < 1 and FMI z-score < 1; Normal muscle-high fat mass: MMI z-score < 1 and FMI z-score ≥ 1; High muscle-normal fat mass: MMI z-score ≥ 1 and FMI z-score < 1; High muscle-high fat mass: MMI z-score ≥ 1 and FMI z-score ≥ 1.

Multivariable-adjusted βs and ORs were also calculated to assess the associations of different body compositions with glucose and lipids. Analyses were conducted using Stata software version 14 (StataCorp, College Station, Texas, USA) and R software version 3.4.0 (R Project for Statistical Computing; www.cran.r-project.org). A 2-tailed *P*<0.05 was considered statistically significant.

## Results

### Participants’ characteristics and prevalence of the dyslipidemia and hyperglycemia

A total of 8,905 participants (4,459 boys [50.1%]) with a mean age of 12.3 ± 3.7 years were included in the present study. **Table 1** shows the prevalence of the dyslipidemia and hyperglycemia with different body compositions according to sex. In the High MMI-High FMI group, participants had the highest prevalence of dyslipidemia, including high TC (6.9% and 6.9%), high TG (22.3% and 6.6%), high LDL-C (8.7% and 7.7%), and low HLD-C (30.9% and 13.4%), which were found in both boys and girls. Girls in the High MMI-High FMI group also had the highest prevalence of hyperglycemia (7.1%), while boys in the normal MMI-High FMI group had the highest prevalence of hyperglycemia (12.3%). In addition, waist and waist-to-height ratio increase as muscle and fat increase. participants with high fat-high muscle have the highest waist and waist-to-height ratio. **Table S1** shows the demographic characteristics.

**Table 1:** Clinical characteristics in boys and girls, stratified by the muscle-fat composition.

Sex	Characteristics	Total	Normal muscle-Normal fat	High muscle-Normal fat	Normal muscle-High fat	High muscle-High fat	*P* ^a^
Boys		(n=4459)	(n=3456)	(n=282)	(n=317)	(n=404)	
	Age	12.1 ± 3.7	12.1 ± 3.7	12.2 ± 3.6	11.9 ± 3.5	12.1 ± 3.7	0.798
	BMI, kg/m^2^	20.1 ± 4.4	18.6 ± 3.0	22.9 ± 3.6	24.4 ± 3.1	27.9 ± 4.4	<0.001
	Waist, cm	68.9 ± 12.8	64.9 ± 9.5	73.8 ± 10.4	81.3 ± 10.8	89.2 ± 12.6	<0.001
	Waist-to-height ratio	0.45 ± 0.06	0.42 ± 0.04	0.47 ± 0.04	0.53 ± 0.04	0.57 ± 0.05	<0.001
	Overweight (including obesity), n (%)	1317 (29.5)	432 (12.5)	200 (70.9)	295 (93.1)	390 (96.5)	<0.001
	Body composition						
	Total fat mass, kg	13.5 ± 7.6	10.8 ± 4.4	15.3 ± 5.5	23.7 ± 6.9	27.3 ± 8.6	<0.001
	Total muscle mass, kg	34.5 ± 13.1	32.6 ± 12	41.8 ± 14.1	35.5 ± 12.1	44.6 ± 15.5	<0.001
	Blood glucose and lipid levels						
	FPG, mmol/L	5.2 ± 0.9	5.2 ± 0.9	5.1 ± 0.9	5.3 ± 1.3	5.4 ± 0.8	0.043
	HOMA-IR, median (IQRs)	1.72(1.20,2.64)	1.69 (1.18,2.54)	1.71 (1.15,2.66)	1.98 (1.24,3)	1.84 (1.27,3.03)	0.111
	Plasma TC, mmol/L	3.79 ± 0.75	3.74 ± 0.72	3.64 ± 0.69	4.08 ± 0.78	4.12 ± 0.86	<0.001
	Plasma TG, mmol/L	0.62(0.44-0.91)	0.58 (0.42,0.80)	0.7 0(0.45,0.98)	0.84 (0.60,1.19)	1.06 (0.67,1.49)	<0.001
	Plasma LDL-C, mmol/L	2.13 ± 0.62	2.06 ± 0.57	2.08 ± 0.58	2.49 ± 0.68	2.53 ± 0.74	<0.001
	Plasma HDL-C, mmol/L	1.38 ± 0.29	1.42 ± 0.29	1.27 ± 0.25	1.28 ± 0.25	1.22 ± 0.23	<0.001
	Plasma non-HDL-C, mmol/L	2.41 ± 0.68	2.32 ± 0.62	2.37 ± 0.62	2.8 ± 0.76	2.91 ± 0.84	<0.001
	Metabolic abnormalities						
	Hyperglycemia	386 (8.7)	288(8.3)	15(5.3)	39(12.3)	44(10.9)	0.006
	Insulin resistance	892(25.2)	632(23.4)	54(24.7)	90(32.4)	116(33.9)	<0.001
	High TC	96 (2.2)	45(1.3)	5(1.8)	18(5.7)	28(6.9)	<0.001
	High TG	252 (5.7)	104 (3.0)	24 (8.5)	34 (10.7)	90 (22.3)	<0.001
	High LDL-C	125 (2.8)	62 (1.8)	6 (2.1)	22 (6.9)	35 (8.7)	<0.001
	Low HDL-C	625 (14.0)	363 (10.5)	62 (22.0)	75 (23.7)	125 (30.9)	<0.001
	High non-HDL-C	94 (2.1)	40 (1.2)	5 (1.8)	19 (6.0)	30 (7.4)	<0.001
Girls		**(n=4446)**	**(n=3527)**	**(n=302)**	**(n=267)**	**(n=350)**	
	Age	12.5 ± 3.7	12.5 ± 3.7	12.2 ± 4.0	12.0 ± 3.8	12.2 ± 3.6	0.007
	BMI, kg/m^2^	19.4 ± 3.7	18.3 ± 2.8	21 ± 3.2	22.7 ± 3.2	25.9 ± 4.1	<0.001
	Waist, cm	64.6 ± 9.9	62.1 ± 7.9	66.8 ± 9.5	72.8 ± 8.7	80.1 ± 10.6	<0.001
	Waist-to-height ratio	0.43 ± 0.05	0.42 ± 0.04	0.45 ± 0.04	0.49 ± 0.04	0.53 ± 0.05	<0.001
	Overweight (including obesity), n (%)	797(17.9)	142(4.0)	115(38.1)	201(75.3)	339(96.9)	<0.001
	Body composition						
	Total fat mass, kg	14.9 ± 6.7	13.3 ± 5.1	15.1 ± 5.8	21.9 ± 6.6	25.4 ± 8.5	<0.001
	Total muscle mass, kg	28.4 ± 8.2	27.3 ± 7.5	32.1 ± 9.1	29.0 ± 8.0	35.8 ± 9.6	<0.001
	Blood glucose and lipid levels						
	FPG, mmol/L	5.0 ± 0.9	5.0 ± 0.9	5.0 ± 1.0	5.2 ± 1.0	5.2 ± 1.1	<0.001
	HOMA-IR, median (IQRs)	1.68(1.12,2.58)	1.67(1.12,2.54)	1.52(0.91,2.33)	1.73(1.26,2.78)	1.83(1.22,3.04)	0.001
	Plasma TC, mmol/L	3.88 ± 0.75	3.87 ± 0.75	3.69 ± 0.73	3.96 ± 0.73	4.04 ± 0.78	0.003
	Plasma TG, mmol/L	0.67 (0.48-0.92)	0.64 (0.47,0.87)	0.69 (0.50,0.96)	0.76 (0.54,1.04)	0.90 (0.64,1.23)	<0.001
	Plasma LDL-C, mmol/L	2.18 ± 063	2.16 ± 0.62	2.07 ± 0.59	2.31 ± 0.62	2.44 ± 0.67	<0.001
	Plasma HDL-C, mmol/L	1.43 ± 0.29	1.46 ± 0.28	1.35 ± 0.26	1.35 ± 0.25	1.28 ± 0.28	<0.001
	Plasma non-HDL-C, mmol/L	2.45 ± 0.67	2.41 ± 0.66	2.35 ± 0.64	2.61 ± 0.69	2.76 ± 0.71	<0.001
	Metabolic abnormalities, n (%)						
	Hyperglycemia	219 (4.9)	162 (4.6)	18 (6.0)	14(5.3)	25 (7.1)	0.154
	Insulin resistance	867 (25.2)	654 (24.1)	45 (20.6)	66 (30.0)	102 (33.9)	<0.001
	High TC	164 (3.7)	125 (3.6)	4 (1.3)	11 (4.1)	24 (6.9)	0.002
	High TG	96 (2.2)	51 (1.5)	11 (3.6)	11 (4.1)	23 (6.6)	<0.001
	High LDL-C	136 (3.1)	96 (2.7)	5 (1.7)	8 (3.0)	27 (7.7)	<0.001
	Low HDL-C	202 (4.5)	118 (3.4)	22 (7.3)	15 (5.6)	47 (13.4)	<0.001
	High non-HDL-C	94 (2.1)	50 (1.4)	2(0.7)	5 (1.9)	18 (5.2)	<0.001

FPG, fasting plasma glucose; HDL-C, high-density lipoprotein cholesterol; HOMA-IR, homeostasis model assessment of insulin resistance; LDL-C, low-density lipoprotein cholesterol; non-HDL-C, non–high-density lipoprotein cholesterol; TC, total cholesterol; TG, triglyceride· Normal fat, fat mass index z score < 1; Normal muscle, muscle mass index z score < 1; High fat, fat mass index z score ≥ 1; High muscle, muscle mass index z score ≥ 1.

Data are presented as means ± SDs or medians (IQRs) for continuous variables and numbers (%) for categorical variables.

a P-value from ANOVA and Kruskal-Wallis test for continuous variables and χ2 test for categorical variables, comparing differences among four groups.

Overweight was defined as BMI greater than sex- and age-specific BMI overweight cutoff values recommended by the International Obesity Task Force.

### Blood glucose and lipid levels at different body composition

**Figure 1:**
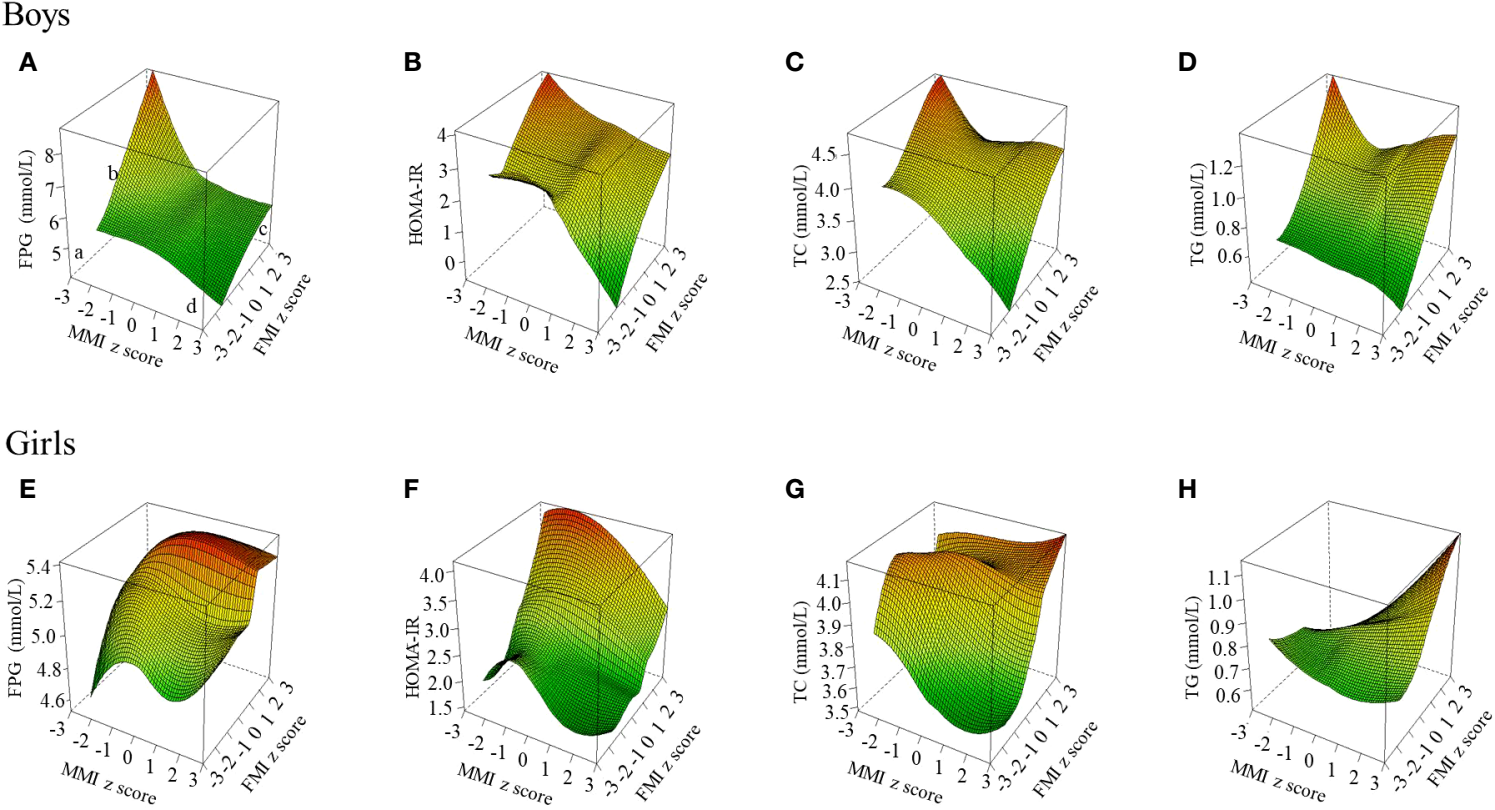
Blood glucose and lipid levels at different muscle-fat composition in boys and girls. The figure shows the results of TC and TG; the other results of blood lipid indicators (LDL-C, HDL-C, and non-HDL-c) are shown in **Figure S1**. FPG, fasting plasma glucose; FMI, fat mass index; HOMA-IR, homeostasis model assessment of insulin resistance; MMI, muscle mass index; TC, total cholesterol; TG, triglyceride. Three-dimensional surface plot of FPG, HOMA-IR, TC, and TG levels according to muscle-fat composition. (a) Low muscle mass and low-fat mass; (b) Low muscle mass and high-fat mass; (c) High muscle mass and high-fat mass; (d) High muscle mass and low-fat mass. Fat mass index and muscle mass index were age- and sex-specific z-score transformed. **(A)** FPG level in boys; **(B)** HOMA-IR level in boys; **(C)** TC level in boys; **(D)** TG level in boys; **(E)** FPG level in girls; **(F)** HOMA-IR level in girls; **(G)** TC level in girls; **(H)** TG level in girls.

**Figure 1** shows the distribution of glucose and main lipids (TC and TG) according to muscle and fat mass. In boys, FPG and TC continued to decrease with increasing muscle mass in low-fat mass level (FMI z-score < 1), but not in high-fat mass (FMI z-score ≥ 1). TG remained at a low level in those with low-fat mass, even when the muscle mass increased. But there was a U-shaped relation between TG and muscle mass in those with high-fat mass. In girls, FPG, TC and TG kept decreasing or remained at a low level with increasing muscle in those with low fat mass (FMI z-score < 1), while kept rising with increasing muscle mass in those with high-fat mass (FMI z-score ≥ 1). And the highest FPG, TC, and TG levels were at C point (the high muscle and high fat mass point). Based on that, we used a z-score of 1 as a cut-off point to stratify fat mass to quantitatively study the associations of muscle mass with glucose and lipids in different fat mass levels, and used z-score of 1 to classify fat and muscle respectively and combine them into the 4 body compositions mentioned in the method to analyze the associations of body composition with glucose and lipids. The distributions of LDL-C and non-HDL-C were same as TC, and can be found in **Figure S1**.

### Associations of muscle mass with glucose and lipids at different fat mass levels

**Figure 2:**
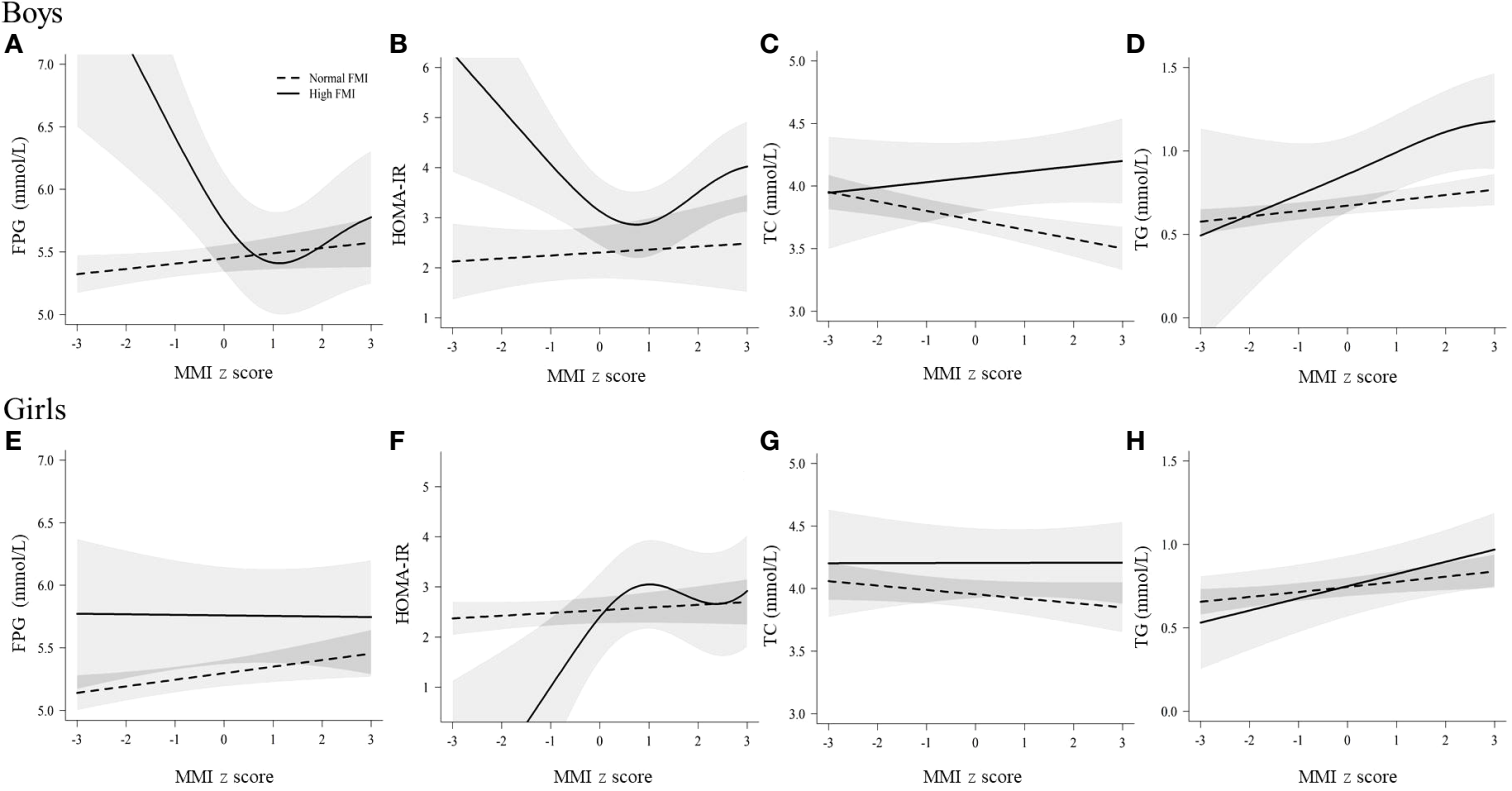
Relations of muscle mass with glucose and lipids in boys and girls, stratified b fat mass levels. The associations of muscle mass with TC and TG are shown; the associations of other blood lipid indicators (LDL-C, HDL-C and non-HDL-c) are shown in **Figure S2**. FPG, fasting plasma glucose; FMI, fat mass index; HOMA-IR, homeostasis model assessment of insulin resistance; MMI, muscle mass index; TC, total cholesterol; TG, triglyceride. Normal FMI: fat mass index z score < 1, High FMI: fat mass index z score ≥ 1. Solid linesdenote high FMI; dotted lines denote normal FMI; shadow parts indicate 95% CIs. The model was adjusted for age, family income, puberty development, physical activity, sedentary activity, drinking alcohol, and smoking status. Fat mass index and muscle mass index were age- and sex-specific z-score transformed. **(A)** The relationship between muscle mass and FPG in boys; **(B)** The relationship between muscle mass and HOMA-IR in boys; **(C)** The relationship between muscle mass and TC in boys; **(D)** The relationship between muscle mass and TG in boys; **(E)** The relationship between muscle mass and FPG in girls; **(F)** The relationship between muscle mass and HOMA-IR in girls; **(G)** The relationship between muscle mass and TC in girls; **(H)** The relationship between muscle mass and TG in girls.

**Figure 2** shows the relations between muscle mass and glucose and main lipids in groups with different fat mass level as derived from the GAM. In the group with normal fat mass (FMI z-score < 1), TC significantly decreased with increasing muscle mass in boys and girls (*P =* 0.015 and 0.017). In the group with high-fat mass (FMI z-score ≥ 1), a significant reversed J-shaped relation between muscle mass and FPG and HOMA-IR (*P =* 0.017 and 0.019) was observed in boys, and TG significantly increased with increasing muscle mass in boys and girls (*P =* 0.026 and 0.020). The relations of muscle mass with LDL-C, HDL-C and non-HDL-C can be found in **Figure S2**, and HDL-C significantly decreased with increasing muscle mass in boys and girls (P < 0.05).

Based on **Figure 2** and **Figure S2**, we first quantitatively analyzed the linear associations of muscle mass with glucose and lipids by fat mass stratification, and **Table 2** shows the adjusted βs and ORs between muscle mass and glucose and lipids in normal fat and high fa group. In normal fat mass group, muscle mass was negatively associated with TC (β = -0.07; 95%CI: -0.12,-0.03; *P <* 0.001) and hyperglycemia (OR = 0.62; 95%CI: 0.46,0.84; *P =* 0.002) in boys; TG significantly increased with increasing muscle mass (β = 0.04; 95%CI: 0.01,0.06; *P =* 0.007) and HDL-C significantly decreased with increasing muscle mass (β = -0.05; 95%CI: -0.07,-0.03; *P <* 0.001) in girls. In high fat mass levels, the negative association between muscle mass and TC was no longer statistically significant in boys with high body fat mass (β = 0.04; 95%CI: -0.05,0.14; *P =* 0.374), and the associations between muscle mass and TG and HDL-c were still the same as in girls with normal body fat mass.

**Table 2:** Adjusted βs and ORs of glucose and lipids associated with muscle mass, stratified by sex and fat mass levels.

Glucose and lipids	Boys						Girls					
	Normal fat			High fat			Normal fat			High fat		
**Parameters**	**β**	**95%CI**	***P***	**β**	**95%CI**	***P***	**β**	**95%CI**	**P**	**β**	**95%CI**	***P***
FPG	-0.04	(-0.13,0.09)	0.052	–			0.05	(0.01,0.09)	0.076	-0.01	(-0.12,0.11)	0.941
HOMA-IR	0.05	(-0.18,0.28)	0.692	–			0.05	(-0.05,0.14)	0.367	–		
TC	-0.07	(-0.12,-0.03)	<0.001	0.04	(-0.05,0.14)	0.374	-0.04	(-0.08,0.01)	0.118	0.01	(-0.08,0.09)	0.987
TG	0.03	(-0.01,0.06)	0.056	0.06	(-0.01,0.12)	0.072	0.04	(0.01,0.06)	0.007	0.08	(0.02,0.14)	0.005
LDL-C	-0.01	(-0.04,0.03)	0.776	0.05	(-0.03,0.13)	0.246	0.01	(-0.04,0.04)	0.979	0.03	(-0.04,0.11)	0.386
HDL-C	-0.08	(-0.09,-0.06)	<0.001	-0.03	(-0.06,-0.01)	0.008	-0.05	(-0.07,-0.03)	<0.001	-0.04	(-0.07,-0.01)	0.012
non-HDL-C	0.01	(-0.04,0.04)	0.965	0.08	(-0.02,0.17)	0.113	0.01	(-0.03,0.05)	0.553	0.04	(-0.04,0.12)	0.344
**Abnormalities**	**OR**	**95%CI**	***P***	**OR**	**95%CI**	***P***	**OR**	**95%CI**	***P***	**OR**	**95%CI**	***P***
Hyperglycemia	0.62	(0.46,0.84)	0.002	0.83	(0.55,1.25)	0.368	1.22	(0.90,1.66)	0.198	1.22	(0.71,2.09)	0.480
Insulin resistance	1.07	(0.90,1.27)	0.450	1.17	(0.89,1.52)	0.261	1.03	(0.87,1.22)	0.733	1.13	(0.87,1.48)	0.361
High TC	0.71	(0.40,1.24)	0.228	0.87	(0.55,1.38)	0.558	1.02	(0.75,1.40)	0.898	1.14	(0.76,1.72)	0.526
High TG	1.77	(0.99,2.44)	0.062	1.61	(1.19,2.18)	0.002	1.48	(0.90,2.42)	0.123	1.19	(0.76,1.85)	0.441
High LDL-C	0.88	(0.53,1.47)	0.624	0.97	(0.64,1.49)	0.903	1.06	(0.74,1.50)	0.755	1.55	(0.98,2.46)	0.062
Low HDL-C	1.56	(1.27,1.92)	<0.001	1.27	(0.98,1.65)	0.073	1.27	(0.90,1.79)	0.172	1.62	(1.11,2.35)	0.011
High non-HDL-C	0.90	(0.51,1.59)	0.723	0.94	(0.61,1.45)	0.776	0.78	(0.50,1.23)	0.286	1.49	(0.91,2.42)	0.113

FPG, fasting plasma glucose; HDL-C, high-density lipoprotein cholesterol; HOMA-IR, homeostasis model assessment of insulin resistance; LDL-C, low-density lipoprotein cholesterol; non-HDL-C, non–high-density lipoprotein cholesterol; TC, total cholesterol; TG, triglyceride.

Normal fat, fat mass index z score < 1; High fat, fat mass index z score ≥ 1.

“-” linear regression analysis cannot be used as **Figure 2** shows the nonlinear association.

Secondly, muscle mass was found to have a non-linear relationship with FPG and HOMA-IR in those with high fat mass in **Figure 2**, so we stratified the muscle mass to study the effect of different muscle mass levels on FPG in high-fat mass by piecewise regression in **Table 3**. Muscle mass was negatively associated with FPG (β = -0.68; 95%CI: -1.19,-0.18; *P =* 0.008) in boys when MMI z-score < 1. However, there was no significant association between high muscle mass (MMI z-score ≥ 1) and hyperglycemia in high fat levels.

**Table 3:** Piecewise regression results of the association between muscle mass and glucose in high fat levels, stratified by sex.

	Boys						Girls					
	Normal muscle			High muscle			Normal muscle			High muscle		
**Parameters**	**β**	**95%CI**	***P***	**β**	**95%CI**	***P***	**β**	**95%CI**	***P***	**β**	**95%CI**	***P***
FPG	-0.68	(-1.19,-0.18)	0.008	0.10	(-0.06,0.27)	0.205	-0.03	(-0.12,0.04)	0.720	0.25	(-0.02,0.45)	0.173
HOMA-IR	0.05	(-0.04,0.14)	0.267	0.01	(-0.22,0.25)	0.925	-0.01	(-0.09,0.07)	0.830	0.39	(-0.01,0.69)	0.160
**Abnormalities**	**OR**	**95%CI**	***P***	**OR**	**95%CI**	***P***	**OR**	**95%CI**	***P***	**OR**	**95%CI**	***P***
Hyperglycemia	0.52	(0.19,1.42)	0.204	1.49	(0.77,2.89)	0.238	0.73	(0.13,1.66)	0.220	1.45	(0.88,1.97)	0.245
Insulin resistance	0.68	(0.32,1.41)	0.300	1.28	(0.78,2.08)	0.330	0.63	(0.26,1.51)	0.300	1.25	(0.76,2.05)	0.388

FPG, fasting plasma glucose; HOMA-IR, homeostasis model assessment of insulin resistance.

Normal muscle, muscle mass index z score < 1; High muscle, muscle mass index z score ≥ 1.

### Associations of body composition with abnormal glucose and lipid metabolism

**Table 4** shows the likelihood of having abnormal glucose and lipid metabolism in relation to different body compositions. Boys with high muscle mass and normal fat mass had a significantly lower risk of hyperglycemia (OR = 0.13, 95%CI: 0.02,0.96, *P =* 0.045) than those without high muscle mass, while boys with high muscle mass and high fat mass had no lower risk of hyperglycemia compared to those with normal mass and normal mass (OR = 1.28; 95%CI: 0.69,2.36, *P =* 0.438). Furthermore, compared with children and adolescents with normal muscle mass and normal fat mass, children and adolescents with high muscle mass and high fat mass had highest risk of insulin resistance, higher TC, higher TG, higher LDL-C, lower HDL-C, and higher non-HDL-C.

**Table 4:** Adjusted ORs (95%CIs) for the associations of muscle-fat composition with glucose and lipids metabolic abnormalities, stratified by sex.

	Boys				Girls			
	OR	95%CI	*P*		OR		95%CI		*P*	
Hyperglycemia									
Normal muscle-Normal fat		Reference					Reference		
High muscle-Normal fat	0.13	(0.02,0.96)	0.045		2.15		(0.96,4.82)		0.064
Normal muscle-High fat	1.94	(1.07,3.52)	0.029		0.98		(0.38,2.53)		0.969
High muscle-High fat	1.28	(0.69,2.36)	0.438		1.27		(0.56,2.88)		0.569
Insulin resistance									
Normal muscle-Normal fat		Reference					Reference		
High muscle-Normal fat	0.96	(0.56,1.64)	0.884		1.32		(0.75,2.32)		0.344
Normal muscle-High fat	1.68	(1.11,2.56)	0.014		1.36		(0.88,2.08)		0.165
High muscle-High fat	2.22	(1.53,3.23)	<0.001		1.66		(1.10,2.51)		0.016
High TC									
Normal muscle-Normal fat		Reference					Reference			
High muscle-Normal fat	1.18	(0.27,5.23)	0.829		0.41		(0.10,1.73)		0.226
Normal muscle-High fat	4.89	(2.04,11.74)	<0.001		1.48		(0.69,3.22)		0.316
High muscle-High fat	6.56	(3.16,13.63)	<0.001		2.38		(1.28,4.43)		0.006
High TG									
Normal muscle-Normal fat		Reference					Reference		
High muscle-Normal fat	2.66	(1.28,5.51)	0.008		2.68		(0.93,7.68)		0.067
Normal muscle-High fat	3.83	(1.97,7.44)	<0.001		5.83		(2.40,14.16)		<0.001
High muscle-High fat	11.11	(6.9,17.91)	<0.001		6.96		(3.06,15.87)		<0.001
High LDL-C									
Normal muscle-Normal fat		Reference					Reference		
High muscle-Normal fat	1.71	(0.49,5.91)	0.399		0.55		(0.13,2.35)		0.419
Normal muscle-High fat	5.36	(2.4,11.96)	<0.001		0.91		(0.32,2.60)		0.858
High muscle-High fat	6.84	(3.43,13.63)	<0.001		2.63		(1.34,5.16)		0.005
Low HDL-C									
Normal muscle-Normal fat		Reference					Reference		
High muscle-Normal fat	2.21	(1.35,3.63)	0.002		1.64		(0.62,4.33)		0.316
Normal muscle-High fat	2.78	(1.74,4.45)	<0.001		2.20		(1.03,4.70)		0.040
High muscle-High fat	4.36	(2.97,6.39)	<0.001		3.95		(2.15,7.25)		<0.001
High non-HDL-C									
Normal muscle-Normal fat		Reference					Reference		
High muscle-Normal fat	1.30	(0.29,5.80)	0.732		0.46		(0.11,1.91)		0.288
Normal muscle-High fat	5.76	(2.37,13.98)	<0.001		1.58		(0.53,4.67)		0.408
High muscle-High fat	8.60	(4.16,17.78)	<0.001		3.55		(1.66,7.61)		0.001

HDL-C, high-density lipoprotein cholesterol; LDL-C, low-density lipoprotein cholesterol; non-HDL-C, non–high-density lipoprotein cholesterol; TC, total cholesterol; TG, triglyceride. Normal fat, fat mass index z score < 1; Normal muscle, muscle mass index z score < 1; High fat, fat mass index z score ≥ 1; High muscle, muscle mass index z score ≥ 1.

## Discussion

The present study found that the prevalence and risk of hyperglycemia and dyslipidemia varied by body composition, with the highest prevalence and risk among those with high muscle mass and high fat mass. We further examined the association of muscle mass with glucose and lipids metabolism stratified by fat mass and found that a negative and significant association of muscle mass with glucose and TC in normal fat boys, but not in high fat boys.

Muscle is an important tissue related to human endocrine and metabolism. The analysis of skeletal muscle proteomics showed that skeletal muscle secretes a variety of myokines, which not only regulate energy demand, but also contribute to cardiovascular, metabolic and mental health and reduce cardiovascular metabolic abnormalities (hyperglycemia, hyperlipidemia, insulin resistance) occur (9, 23). Few pediatric studies have investigated the association of muscle mass with cardiometabolic risk, especially by weight status, and the results are partially consistent with ours. A study of Chilean children without obesity showed that high muscle mass was associated with lower values of metabolic syndrome z-score and TC (24). Among adolescents from the National Health and Nutrition Examination Survey, non-Hispanic blacks had a significantly lower prevalence of metabolic syndrome (2.5%) than Mexican Americans (12.9%) and non-Hispanic whites (10.9%), and in this population of adolescents, non-Hispanic blacks had a higher lean mass index than non-Hispanic whites and Mexican Americans in same weightstatus (25). Recent evidence suggests that muscle fitness levels in school-aged children and adolescents are declining (26, 27), and the World Health Organization supports the addition of muscle-strengthening activities in addition to aerobic exercise as part of physical activity guidelines for children and adolescents and suggests that monitoring muscle fitness can support Development of health promotion strategies (28, 29). Therefore, accurate assessment and monitoring of muscle development and health levels in children and adolescents is an important basis for scientific prevention and control of obesity and reduction of cardiovascular abnormalities.

Different from previous study found that muscle mass have a protective effect on cardiovascular health in children with obesity defined by BMI (24), the additional and important new insight from the present study is that muscle mass was not associated with hyperglycemia and TC in boys with high fat mass. The reason for the difference may be related to the inability of BMI to accurately assess the body composition, and there may be high muscle and low fat phenotype in children and adolescents with obesity evaluated by BMI, which affect the results of the association between muscle and cardiometabolic risk in children and adolescents with obesity (30). Furthermore, high muscle mass in the context of high fat mass may more mean the presence of excess fat in the muscle, and ectopic fat accumulation in muscle has previously been reported in children and adolescents with obesity (31, 32). Small ectopic fat depots in muscle that can adversely affect muscle function, as studies confirmed that immune cells in intramyocellular/intermuscular adiposity tissue in obesity tend to negatively regulate myocyte metabolic functions (16), and a study of 236 Finnish girls also showed that ectopic fat accumulation in skeletal muscle were important factors in increased cardiometabolic risk in adulthood (33).

Consistent with adults’ studies indicated that low muscle mass and high fat mass might have an important role in diabetes and cardiovascular diseases (34, 35), we found that normal muscle and high fat mass had higher prevalence of hyperglycemia and hyperlipidemia. Moreover, present results showed that compared with normal fat-normal muscle group, high fat-high muscle group had the highest risk of abnormal glucose and lipid metabolism. This may be related to the highest levels of waist and waist-to-heigh ratio in high fat-high muscle participants, previous studies have showed that Asians, including Chinese, are more likely to accumulate fat in the abdomen, which is associated with a higher risk of cardiometabolic abnormalities (36).

Many previous studies on young people, including ours, mainly studied the effect of fat mass on cardiometabolic risk (37–40). Some of them have demonstrated that fat stored in different regions has a differential impact on cardiometabolic risk in youth (39, 40), and pointed to that as one of the mechanisms for obesity heterogeneity. Our results extended the mechanisms of obese heterogeneity within the context of a large population-based sample of Chinese children and adolescents, and we found that different body compositions had a different impact on cardiometabolic risk in young individuals, suggesting that body composition may also be important for identifying cardiometabolic risk in children and adolescents.

The underlying sex differences in the fat mass modifying effect of muscle mass on glucose and lipids metabolism are unclear. Nevertheless, fat has been shown to adversely affect the endocrine system, including skeletal muscle, with gender differences (16, 41). Preclinical studies have shown that high fat can reduce the effect of muscle secretion of adiponectin, which has also been recently described as a muscle cytokine (42, 43). As a pleiotropic hormone, adiponectin very effectively enhances insulin sensitivity and decreases glucose (44). We analyzed 2085 samples containing adiponectin data and found that the boys with high muscle mass and high fat mass had the lowest adiponectin levels, but the girls with high muscle mass and high fat mass had the nearly highest levels (**Figure S3**). This also suggested that body composition might affect the endocrine function and there are gender differences.

The strength of the study is the large sample study recruited in schools from seven provinces of China, thus having a high mathematical power. Body composition and biochemical metrics were obtained using the gold standard techniques of DXA and HOMA protocols rather than peripheral measures, such as BMI and waist. Furthermore, this study is focused on ethnic-geographic regions, as it is one of the rare contemporary studies to address body compositions and glucose and lipid metabolism in the populations of Asian mega-cities, especially in mainland China. However, several limitations should be paid attention when interpreting the findings. First, the delay between the end of data collection and article submission may compromise the contribution of the findings to contemporary scientific and social representation, as the current population may have genotypic and phenotypic differences from the population at the time of data collection. Second, nutritional habits, physical activity, menstrual cycle and other unknown factors may account the associations observed in this study, particularly in the association of muscle with biochemical indicators. As children who met daily recommended moderate to vigorous physical activity or adhered to a Mediterranean diet were found to have more favorable blood lipids and body composition variables levels compared to those who did not meet these recommendations, and irregular menstrual cycles were considered risk factors for the development of metabolic disorders (45–48). Third, muscle can be measured in terms of mass and quality. The muscle quality and aerobic levels needs to be elucidated, which may help to better understand our observed associations of muscle with abnormal glucose and lipid metabolism risk. Finally, this cross-sectional study had a limited ability to demonstrate causal relationships between body interaction and glucose and lipids metabolism.

In conclusion, the prevalence of hyperglycemia and dyslipidemia was highest in children and adolescents with high muscle mass and high fat mass, and the associations of muscle mass with glucose and lipid metabolism were modified by fat mass in children and adolescents. These results demonstrate that body composition plays an important role in explaining the heterogeneity of the association between obesity and cardiometabolic risk. Specifically, it highlights the importance of focusing on muscle when studying the effects of obesity on the cardiometabolic risk factors, and high muscle mass in high-fat children and adolescents may not imply a reduction in cardiometabolic risk. Prospective studies are warranted to evaluate the long-term impacts of body composition on cardiovascular risk.

## Group members

The investigators of the China Child and Adolescent Cardiovascular Health collaboration group are listed in the Supplemental Text S1.

## Data availability statement

The original contributions presented in the study are included in the article/**Supplementary Material**. Further inquiries can be directed to the corresponding author.

## Ethics statement

The studies involving human participants were reviewed and approved by the ethics committee of the Capital Institute of Pediatrics (approval number: 2012062). Written informed consent to participate in this study was provided by the participants’ legal guardian/next of kin.

## Author contributions

JM had full access to all the data in the study and takes responsibility for the integrity of the data, conceptualization, and design of the study; she reviewed the manuscript and had primary responsibility for the final content; LG and HC conducted all analyses, interpreted the results, and drafted the manuscript; JL, XS, and YY were involved in data acquisition; XW contributed to the discussion. All authors reviewed the manuscript and had final approval of the submitted and published versions.

## Funding

This study was supported by the National Natural Science Foundation of China (grant number: 81973110) and the National Science and Technology Support Projects for the “Twelfth Five-Year Plan” of China (2012BAI03B03).

## Acknowledgments

We thank Haibo Li from Fujian Maternity and Child Health Hospital, and Liwan Fu and Pei Xiao from Beijing Children’s Hospital for their assistance in editing figures. Data described in the manuscript, codebook, and analytic code will be made available from the corresponding author on reasonable request.

## Conflict of interest

The authors declare that the research was conducted in the absence of any commercial or financial relationships that could be construed as a potential conflict of interest.

## Publisher’s note

All claims expressed in this article are solely those of the authors and do not necessarily represent those of their affiliated organizations, or those of the publisher, the editors and the reviewers. Any product that may be evaluated in this article, or claim that may be made by its manufacturer, is not guaranteed or endorsed by the publisher.
