# Supplementary material for: The associations of muscle mass with glucose and lipid metabolism are influenced by body fat accumulation in children and adolescents
Source: Front Endocrinol (Lausanne). 2022 Sep 15;13:976998. doi: 10.3389/fendo.2022.976998 (PMC9520779; doi:10.3389/fendo.2022.976998)
Supplement: Supplementary file 1 [file DataSheet_1.pdf]

Supplementary materials

The effect of muscle mass on glucose and lipid metabolism is influenced by body fat accumulation in children

Liwang Gao [1#], Hong Cheng [2#], Yinkun Yan [1], Junting Liu [2], Xinying Shan [2], Xi Wang [1], Jie Mi [1,2*], on behalf of the China Child and Adolescent Cardiovascular Health (CCACH) Collaboration Group [†]

[1] Center for Non-communicable Disease Management, Beijing Children's Hospital, Capital Medical University, National Center for Children's Health, Beijing 100045, China;
[2] Department of Epidemiology, Capital Institute of Pediatrics, Beijing 100020, China;

[#] Liwang Gao and Hong Cheng are equal contribution

[†] The investigators of the China Child and Adolescent Cardiovascular Health collaboration group are listed in the Supplemental Text S1.

[*] Correspondence: Address to Jie Mi, MD, PhD, Center for Non-communicable Disease Management, Beijing Children's Hospital, Capital Medical University, National Center for Children's Health, No. 56 Nanlishi Road, Xicheng District, Beijing 100045, China; Department of Epidemiology, Capital Institute of Pediatrics, No. 2 Yabao Road, Chaoyang District, Beijing 100020, China. E-mail: jiemi12@vip.sina.com.

Text S1. List of investigators of the China Child and Adolescent Cardiovascular Health

Jie Mi, MD, PhD, Yinkun Yan, MD, PhD, Hongbo Dong, MD, PhD, Center for Non-communicable Disease Management, Beijing Children’s Hospital, Capital Medical University, National Center for Children’s Health, Beijing, China;

Junting Liu, MS, Xiaoyuan Zhao, MS, Hong Cheng, MS, Dongqing Hou, MS, Fangfang Chen, MS, Guimin Huang, MS, Linghui Meng, PhD, Qin Liu, MS, Meixian Zhang, PhD, Wenpeng Wang, MS, Lijun Wu, PhD, Xinying Shan, MS, Ping Yang, MS, Department of Epidemiology, Capital Institute of Pediatrics, Beijing, China;

Jinghui Sun, MD, Xiaona Wang, MD, Pediatric Cardiovascular Department, The First Hospital of Jilin University, Changchun, Jilin, China;

Ying Li, MD, Department of Pediatrics, People’s Hospital of Tonghua, Tonghua, Jilin, China;

Weili Yan, PhD, Fang Liu, PhD, Yi Zhang, MPH, Yi Cheng, MS, Shaoke Chen, MS, Qian Zhao, MS, Fang Cao, MS, Kai Mu, MS, Dayan Niu, MS, Department of Clinical Epidemiology, Children’s Hospital of Fudan University, Shanghai, China;

Bo Xi, PhD, Min Zhao, PhD, Department of Epidemiology, School of Public Health, Shandong University, Jinan, Shandong, China;

Feng Xiong, MD, Gaohui Zhu, PhD, Department of Endocrinology, Children’s Hospital of Chongqing Medical University, Chongqing, China;

Lan Zhang, MD. Guidance Center of Child Health Care, Chengdu Women's and Children's Central Hospital, Chengdu, Sichuan, China;

Meng Mao, MD. Department of Child Health Care, West China Second University Hospital, Chengdu, Sichuan, China;

Wenqing Ding, PhD, Ling Zhang, PhD, Leina Jia, MD, Department of Health Education and Child Health Care, School of Public Health, Ningxia University, Ningxia, China.

Table S1. General characteristics in boys and girls, stratified by muscle-fat composition

Sex	Characteristics	Total	Normal muscle-nromal fat	High muscle-normal fat	Normal muscle-high fat	High muscle-high fat	*P* [a]
**Boys**							
	Family income ($)						0.519
	< 9400	1457(32.7)	1116(32.3)	87(30.9)	114(36.0)	140(34.7)	
	9400 - 23999	776(17.4)	587(17.0)	57(20.2)	54(17.0)	78(19.3)	
	≥ 24000	343(7.7)	269(7.8)	24(8.5)	25(7.9)	25(6.2)	
	Spermarche	622(13.9)	503(14.6)	29(10.3)	37(11.7)	53(13.1)	0.037
	Physical inactivity (> 1h/d)	574(12.9)	443(12.8)	42(14.9)	29(9.1)	60(14.9)	0.095
	Sedentary behavior (< 2h/d)	1890(42.4)	1464(42.4)	118(41.8)	142(44.8)	166(41.1)	0.368
	Smoking	526(11.8)	425(12.3)	36(12.8)	22(6.9)	43(10.6)	0.022
	Drinking	1107(24.8)	869(25.1)	70(24.8)	70(22.1)	98(24.3)	0.476
**Girls**							
	Family income ($)						0.670
	< 9400	1609(36.2)	1251(35.5)	117(38.7)	118(44.2)	123(35.1)	
	9400 - 23999	747(16.8)	583(16.5)	44(14.6)	46(17.2)	74(21.1)	
	≥ 24000	307(6.9)	244(6.9)	19(6.3)	20(7.5)	24(6.9)	
	Menarche	2124(47.8)	1692(48.0)	127(42.1)	129(48.3)	176(50.3)	0.095
	Physical inactivity (> 1h/d)	369(8.3)	297(8.4)	23(7.6)	18(6.7)	31(8.9)	0.742
	Sedentary behavior (< 2h/d)	1909(42.9)	1488(42.2)	144(47.7)	127(47.6)	150(42.9)	0.461
	Smoking	207(4.7)	158(4.5)	15(5.0)	8(3.0)	26(7.4)	0.050
	Drinking	827(18.6)	667(18.9)	53(17.5)	41(15.4)	66(18.9)	0.299

Nromal fat, fat mass index z score < 1; Normal muscle, muscle mass index z score < 1; High fat, fat mass index z score ≥ 1; High muscle, muscle mass index z score ≥ 1.

Data are presented as numbers (%).

[a] *P* value from $\chi 2$ test for categorical variables, comparing differences among four groups.

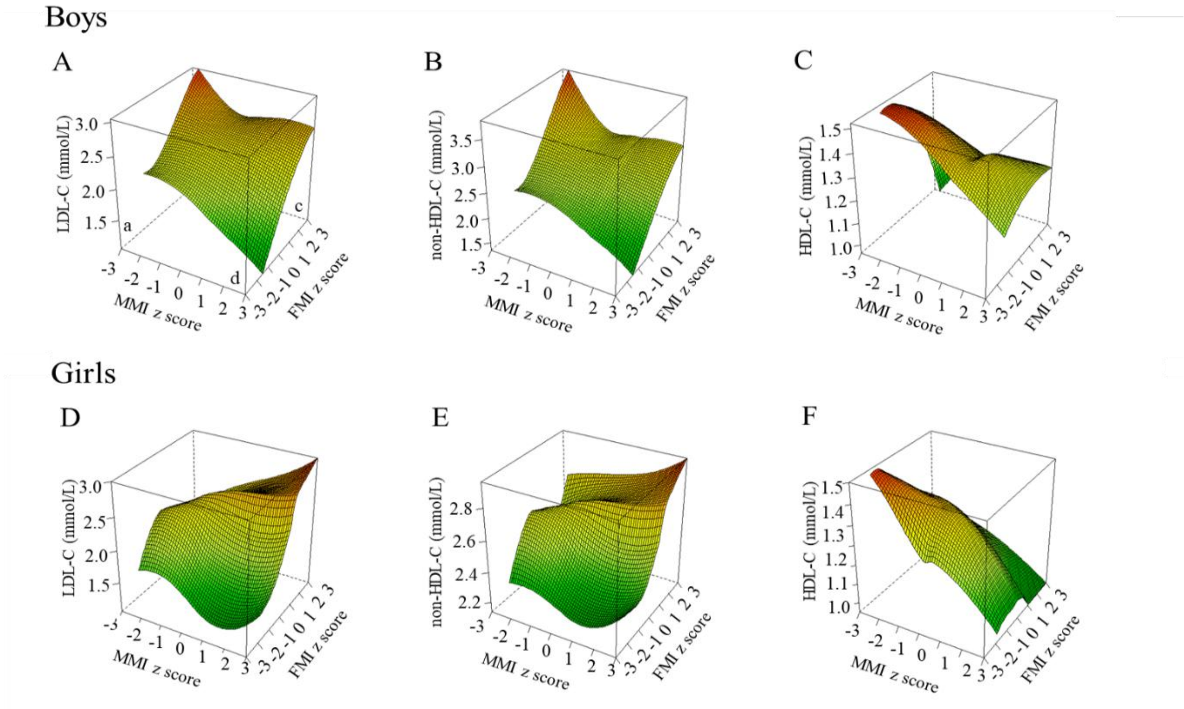

Figure S1 LDL-C, HDL-C, and non-HDL-c levels at different muscle-fat compositions in boys and girls. FMI, fat mass index; HDL-C, high-density lipoprotein cholesterol; LDL-C, low-density lipoprotein cholesterol; MMI, muscle mass index; non-HDL-C, non–high-density lipoprotein cholesterol. Three-dimensional surface plot of LDL-C, HDL-C, and non-HDL-C levels according to muscle-fat composition. (a) Low muscle mass and low-fat mass; (b) Low muscle mass and high-fat mass; (c) high muscle mass and high-fat mass; (d) high muscle mass and low-fat mass. Fat mass index and muscle mass index were age- and sex-specific z-score transformed.

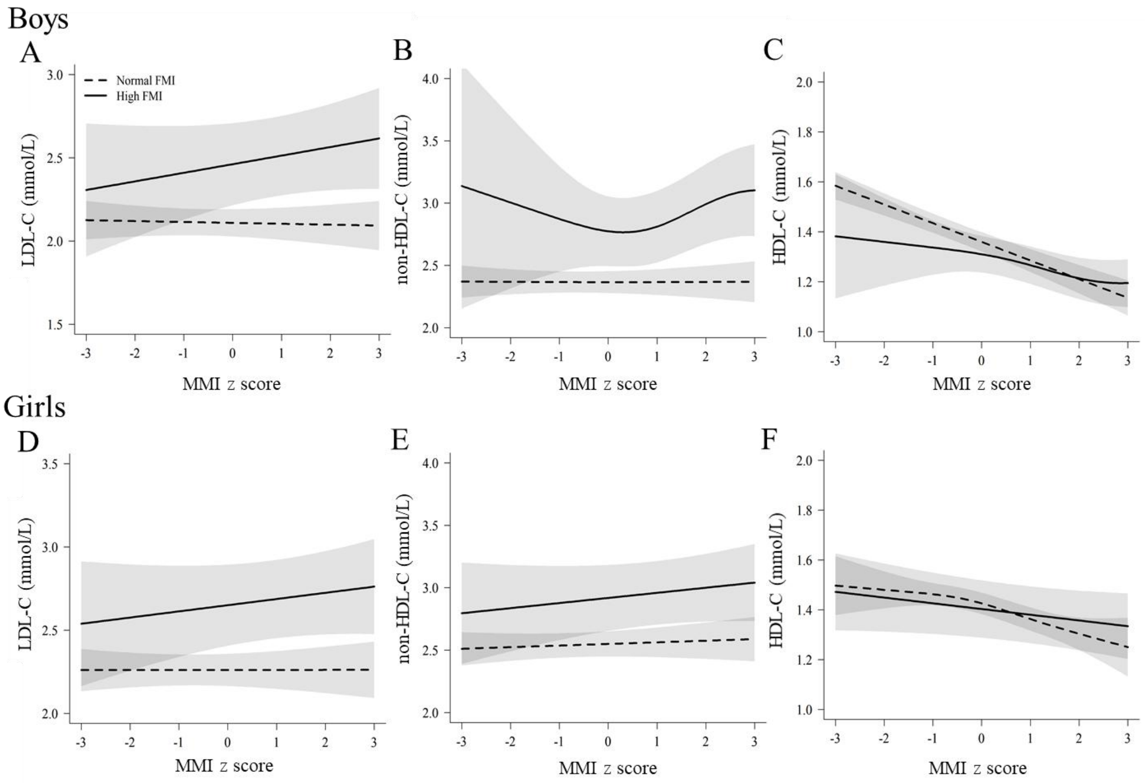

Figure S2 Relations of muscle mass with LDL-C, HDL-C, and non-HDL-c in boys and girls, stratified by fat mass levels. FMI, fat mass index; HDL-C, high-density lipoprotein cholesterol; LDL-C, low-density lipoprotein cholesterol; MMI, muscle mass index; non-HDL-C, non–high-density lipoprotein cholesterol. Normal FMI: fat mass index z score < 1; high FMI: fat mass index z score ≥ 1. Solid lines denote high FMI; dotted lines denote normal FMI; shadow parts indicate 95% CIs. Model adjusted for age, family income, puberty development, physical activity, sedentary activity, drinking alcohol, smoking status. Fat mass index and muscle mass index were age- and sex-specific z-score transformed.

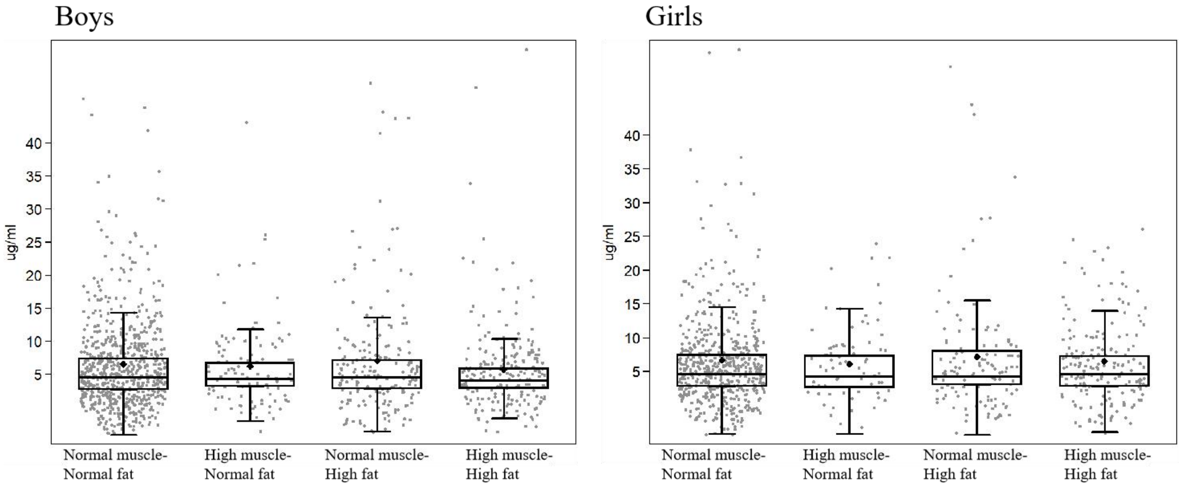

	Normal muscle-Normal fat	High muscle-Normal fat	Normal muscle-High fat	High muscle-High fat
boys	(n=677)	(n=98)	(n=190)	(n=216)
	4.48(2.74,7.37)	4.29(3.17,6.65)	4.51(2.82,7.14)	3.95(2.84,5.84)
girls	(n=528)	(n=74)	(n=119)	(n=183)
	4.55(2.81,7.51)	4.23(2.69,7.24)	4.15(3.08,8.06)	4.52(2.77,7.27)

Figure S3 Medians (IQRs) of adiponectin under different muscle-fat compositions. A total of 2085 samples containing adiponectin data were analyzed. Normal fat, fat mass index z score < 1; Normal muscle, muscle mass index z score < 1; High fat, fat mass index z score ≥ 1; High muscle, muscle mass index z score ≥ 1. Box plots and means (black diamond) show the distribution of adiponectin under different muscle-fat compositions. The table shows the values of the median and quartiles under different muscle-fat compositions.
